# Prostaglandin E2 Stimulates the Expansion of Regulatory Hematopoietic Stem and Progenitor Cells in Type 1 Diabetes

**DOI:** 10.3389/fimmu.2018.01387

**Published:** 2018-06-19

**Authors:** Moufida Ben Nasr, Francesca D’Addio, Amir Mohammad Malvandi, Silvia Faravelli, Eduardo Castillo-Leon, Vera Usuelli, Francesca Rocchio, Teresa Letizia, Abdel Basset El Essawy, Emma Assi, Chiara Mameli, Elisa Giani, Maddalena Macedoni, Anna Maestroni, Alice Dassano, Cristian Loretelli, Moira Paroni, Giuseppe Cannalire, Giacomo Biasucci, Marco Sala, Alessandra Biffi, Gian Vincenzo Zuccotti, Paolo Fiorina

**Affiliations:** ^1^Nephrology Division, Boston Children’s Hospital, Harvard Medical School, Boston, MA, United States; ^2^International Center for T1D, Pediatric Clinical Research Center Fondazione Romeo ed Enrica Invernizzi, Department of Biomedical and Clinical Science L. Sacco, University of Milan, Milan, Italy; ^3^Medicine, Al-Azhar University, Cairo, Egypt; ^4^Department of Pediatrics, Buzzi Children Hospital, Milan, Italy; ^5^Pediatric Clinical Research Center Fondazione Romeo ed Enrica Invernizzi, Department of Biomedical and Clinical Science L. Sacco, University of Milan, Milan, Italy; ^6^Department of Pediatrics, Children’s Hospital Buzzi, Milan, Italy; ^7^Department of Pediatrics, Diabetes Service Studies, University of Milan, Ospedale dei Bambini Vittore Buzzi, Milan, Italy; ^8^Department of Bioscience, University of Milan, Milan, Italy; ^9^Department of Pediatrics and Neonatology, Ospedale Guglielmo da Saliceto, Piacenza, Italy; ^10^Department of Pediatrics, Tradate Hospital, Tradate, Italy; ^11^Gene Therapy Program, Dana-Farber/Boston Children’s Cancer and Blood Disorders Center, Boston, MA, United States; ^12^Harvard Medical School, Boston, MA, United States; ^13^Division of Endocrinology, ASST Sacco Fatebenefratelli-Sacco, Milan, Italy

**Keywords:** hematopoietic stem and progenitor cells, prostaglandins, autoimmune diseases, PD-L1, CXCR4

## Abstract

Hematopoietic stem and progenitor cells (HSPCs) are multipotent stem cells that have been harnessed as a curative therapy for patients with hematological malignancies. Notably, the discovery that HSPCs are endowed with immunoregulatory properties suggests that HSPC-based therapeutic approaches may be used to treat autoimmune diseases. Indeed, infusion with HSPCs has shown promising results in the treatment of type 1 diabetes (T1D) and remains the only “experimental therapy” that has achieved a satisfactory rate of remission (nearly 60%) in T1D. Patients with newly diagnosed T1D have been successfully reverted to normoglycemia by administration of autologous HSPCs in association with a non-myeloablative immunosuppressive regimen. However, this approach is hampered by a high incidence of adverse effects linked to immunosuppression. Herein, we report that while the use of autologous HSPCs is capable of improving C-peptide production in patients with T1D, *ex vivo* modulation of HSPCs with prostaglandins (PGs) increases their immunoregulatory properties by upregulating expression of the immune checkpoint-signaling molecule PD-L1. Surprisingly, CXCR4 was upregulated as well, which could enhance HSPC trafficking toward the inflamed pancreatic zone. When tested in murine and human *in vitro* autoimmune assays, PG-modulated HSPCs were shown to abrogate the autoreactive T cell response. The use of PG-modulated HSPCs may thus provide an attractive and novel treatment of autoimmune diabetes.

## Introduction

Encouraging results of previous pilot trials suggest that autologous hematopoietic stem and progenitor cell transplantation (AHSCT) may be a relevant alternative therapeutic option to immunosuppressive drugs in the treatment of several refractory autoimmune disorders (1, 2). Over 3,000 transplants using AHSCT have been performed worldwide with a very high safety profile (2, 3). We recently demonstrated that AHSCT could induce long-term, drug free, and symptoms-free remission in patients newly diagnosed with type 1 diabetes (T1D). Insulin independence was achieved in nearly 60% of treated subjects at 6 months, with 40% showing sustained insulin-free remission over 4 years following the procedure (4). The aim behind the use of AHSCT is to suppress autoreactive immune cells, while allowing for *de novo* generation of a naïve immune compartment tolerant to pancreatic β cells antigens (5), thus preventing T cell infiltration into targeted organs (6). AHSCT trials showed that in treated patients, an overall resetting of the immune system toward a “regulatory”-like T cell landscape was evident, with an increase in CD4^+^Foxp3^+^ Tregs (7). Unfortunately, the use of immunosuppression during AHSCT limits the potential use of this therapy in T1D to experimental conditions, due to patients’ potential exposure to adverse effects. Interestingly, the immunoregulatory properties of hematopoietic stem and progenitor cells (HSPCs) seem to be linked to their expression of the immune checkpoint-signaling molecule PD-L1 (or CD274) (8, 9). They further express CXCR4, which allows HSPCs to traffic to inflamed area/sites of injuries (10). Unlike mesenchymal or embryonic stem cells, which are associated with the potential development of tumorogenesis and formation of ectopic tissue (5, 11–13), HSPCs have been safely used for years (14–16). Several studies suggested that prostaglandin E2 (PGE2) might have anti-inflammatory effects through inhibition of several pro-inflammatory cytokines (17). Other investigators have demonstrated that the endogenous anti-inflammatory role of PGE2 is mainly mediated through it receptor EP4, thereby inhibiting macrophage derived pro-inflammatory chemokines production during atherogenesis (18, 19). While others have mainly studied in depth the mechanism by which PGE2 can control inflammation and demonstrated that PGE2 plays its regulatory role by limiting T cell activation thereby impairing T cell arrest and inhibiting T cells interactions with dendritic cells (DCs) (20). Previous reports have introduced and identified prostaglandins (PGs) as potentials HSPCs enhancing candidates capable of inducing/improving their long-term maintenance and engraftment faculties (21). We hypothesize that enhancing the immunoregulatory properties of HSPCs using pharmacological modulation with small molecules may create a novel powerful immunoregulatory tool for the treatment of T1D.

## Materials and Methods

### Human Studies

#### Study Population Included in the AHSCT Clinical Trial

Two cohorts consisting of 36 T1D patients were enrolled in the AHSCT program and were also enrolled in three independent clinical trials as previously described (6). Autoantibodies were analyzed on serum by RIA (for insulin autoantibodies) and ELISA (for insulinoma-2-associated autoantibodies, glutamic acid decarboxylase autoantibodies, and Znt8) according to the standard of care clinical procedure. The study was performed in accordance with Institutional Review Board committee approval of each participant Institution, informed consent was provided by all individuals. All baseline demographic and clinical characteristics of the study population are reported in Table 1.

**Table 1:** Baseline demographic and clinical characteristics of patients with T1D treated with autologous non-myeloablative hematopoietic stem cell transplantation in two AHCST cohorts.

Patient characteristics	
Number of patients included	*n* = 36
Age (years ± SEM)	22.4 ± 0.9
Gender (M/F)	27/9
BMI (kg/m^2^ ± SEM)	20.7 ± 0.5
HbA1c (mmol/mol ± SEM)	86.6 ± 6.4
C-peptide (ng/ml ± SEM)	0.73 ± 0.06
Autoantibodies	(% of patients)
*GAD*	86
*Other (IAA, IA-2A, ICA)*	17
DKA or DK history	(% of patients)
*No DKA/DK*	67
*DKA*	28
*DK*	5

*T1D, type 1 diabetes; AHSCT, autologous hematopoietic stem and progenitor cell transplantation; BMI, body mass index; GAD, glutamic acid decarboxylase autoantibodies; ICA, islet cell cytoplasmic autoantibodies; IA-2A, insulinoma-2-associated autoantibodies; IAA, insulin autoantibodies; DKA, diabetic ketoacidosis; DK, diabetic ketosis*.

#### Study Population Included in the PG-Library Screening

Blood samples were obtained from long lasting T1D patients (*n* = 24) and healthy controls (CTRL) (*n* = 5) in accordance with Institutional Review Board committee approval of San Raffaele Hospital and of Boston children’s Hospital (BCH 3851); informed consent was provided by all individuals included in the present study. Baseline characteristics of the study population are summarized in Table 2. Peripheral blood mononuclear cells (PBMCs) isolated from 20 ml blood samples using Lymphoprep (Stem Cell Technologies, Cambridge, MA, USA) were frozen in freezing medium (RPMI 1640 20% FBS and 8% DMSO) and stored at −80°C. After thawing, PBMCs were recovered in culturing medium consisting of RPMI 1640 (Life Technologies, Carlsbad, CA, USA) supplemented with 10% FBS, 2 mM l-glutamine (Life Technologies), 100 U/ml penicillin (Life Technologies), for 48 h, and CD34^+^ cells were then isolated using a CD34 Positive Isolation Kit (Miltenyi Biotec, San Diego, CA, USA) according to the manufacturer’s instructions.

**Table 2:** Clinical characteristics of patients with T1D and of healthy controls included in the PGs library screening.

Patient characteristics	
Number of patients included	*n* = 24
Age (years ± SEM)	58.2 ± 11.6
Gender (M/F)	16/8
BMI (kg/m^2^ ± SEM)	20.7 ± 0.5
EIR (UI)	18.3 ± 5.4
Concomitant treatment	Levothyroxine (*n* = 8), statin (*n* = 5)

**Healthy control characteristics**	

Number of individuals included	*n* = 5
Age (years ± SEM)	40.8 ± 6.4
Gender (M/F)	2/3

*T1D, type 1 diabetes; BMI, body mass index; EIR, exogenous insulin requirement units; PGs, prostaglandins*.

#### Pharmacological Modulation of Human CD34^+^ Cells

1 × 10^5^ of isolated human CD34^+^ HSPCs (purity 99%) were cultured in 200 µl of StemSpan SFEM II media (SEMCELL Technologies Inc., Cambridge, MA, USA), and each compound in the Prostaglandin Screening Library II (Cayman Chemicals, Ann Arbor, MI, USA), the detailed composition of the library is shown in Table S1 in Supplementary Material, was added individually at day 0 and at day 1 at a concentration of 10 µM as previously reported by our group and others (9, 21). In another assay, isolated CD34^+^ cells from freshly isolated human PBMCs or from cryopreserved PBMCs, and processed as described earlier, were cultured in the presence of a cocktail of cytokines containing: 10 µg/ml heparin (SEMCELL Technologies Inc., Cambridge, MA, USA), 10 ng/ml human stem cell factor (SCF) (Miltenyi Biotec, San Diego, CA, USA), 20 ng/ml human thrombopoietin (TPO) (Miltenyi Biotec, San Diego, CA, USA), 10 ng/ml human FGF-1 (Miltenyi Biotec, San Diego, CA, USA), 100 ng/ml insulin-like growth factor-binding protein 2 (IGFBP2) (R&D Systems, Inc., Minneapolis, MN, USA), and 500 ng/ml angiopoietin-like 3 (R&D Systems, Inc., Minneapolis, MN, USA). PGE2 (PromoKine, PromoCell Gmbh, Germany) was added by pulsing the culture at 0, 24, 72 h and 6 days with 2 µl of diluted PGE2 (10 µM). Cells were cultured for 7 days at 37°C in 5% CO_2_, and CD34^+^ cells were then subjected to FACS analysis and were run on a FACSCelesta™ (Becton Dickinson, Franklin Lakes, NJ, USA). Data were analyzed using FlowJo software version 8.7.3 (Treestar, Ashland, OR, USA). The different cytokines used here and their related concentration as well as the choice of the incubation timing was used as previously reported in the literature (22).

#### Quantitative Reverse Transcriptase Polymerase Chain Reaction (qRT-PCR)

RNA was extracted from CD34^+^ cells using Direct-zol™ RNA Kits (Zymo, Irvine, CA, USA) and Trizol Reagent (Invitrogen Carlsbad, CA, USA), RNA quality was assessed by Multiskan™ GO Microplate spectrophotometer and the ratios of absorbance at 260 and 280 nm were assessed for all the samples. Only samples with RNA ratios within 1.9 were included in the present study. cDNA synthesis was made from purified total RNA by reverse transcription using High capacity cDNA Reverse Transcription RETROscript^®^ Kit (Thermo Fisher Scientific, Waltham, MA, USA) followed by a pre-amplification using Taqman PreAmp Kit (Applied Biosystems) according to the manufacturer’s instructions. qRT-PCR analysis was performed using TaqMan assays (Life Technologies, Grand Island, NY, USA) containing PCR primers and TaqMan probes according to the manufacturer’s instructions. Normalized expression values were determined using the ΔCt method. qRT-PCR data were normalized for the expression of GAPDH. qRT-PCR reactions were performed in triplicate in a 96-well format using an Applied Biosystems 7900HT fast real-time PCR instrument. Relative expression was calculated using the comparative threshold cycle method as previously described (23, 24). For two-groupcomparisons, a Student’s *t* test was employed. Reported below are the main characteristics of the primers used:

**Table:** 

Gene symbol	Assay ID	Refseq accession #	Band size (bp)	Reference position
CD274 (PD-L1)	Hs01125299_m1	NM_ 001267706.1	89	441
CD184 (CXCR4)	Hs00237052_m1	NM_ 001008540.1	153	973
IDO1	Hs00984148_m1	NM_ 0022164.5	66	651
GAPDH	Hs99999905_m1	NM_001289746.1	122	229

#### Human ELISPOT Assay

An ELISPOT assay was used to measure the number of IFN-γ-producing cells according to the manufacturer’s protocol (BD Biosciences, San Jose, CA, USA) as previously shown by our group (2). 1 × 10^6^ PBMCs isolated from T1D patients were cultured for 2 days in the presence of IA-2 peptide (Thermo Fisher Scientific Gmbh, Germany) (100 µg/ml) in RPMI medium supplemented with 10% FBS. At 24 h after stimulation, 500 µl of medium was added to the culture. Cells were collected at day 2 and added to plates coated with anti-IFN-γ antibody (eBioscience, Thermo Fisher Scientific, Waltham, MA, USA) with or without PGE2-modulated CD34^+^ cells at ratios of 1:2 or 1:10 or 1:32 in RPMI medium supplemented with 10% FBS. Spots were counted using an A.EL.VIS Elispot Reader (A.EL.VIS GmbH, Hannover, Germany) or on an Immunospot Reader (C.T.L. Cellular Technology Ltd., Cleveland, OH, USA).

#### Immunofluorescence and Confocal Microscopy

Regulatory CD34^+^ (PGE2-modulated) cells and unmodulated CD34^+^ cells isolated from peripheral blood of healthy controls were fixed in 4% PFA for 1 h at 4 C, washed three times for 20 min in PBS, and cells were counterstained with blue fluorescent DAPI (1:10,000, BioLegend, San Diego, CA, USA) and anti-human PD-L1 (BD Biosciences). Cells were photographed under a 63× objective. Images were captured on a Leica SP5X system with an upright DM6000 microscope and A1R confocal microscope (Nikon Instruments, Melville, NY, USA). Histology was evaluated by at least two expert pathologist (9).

#### Migration Assay

Transwell migration assays were performed on PGE2-modulated HSPCs compared to unmodulated HSPCs in the presence of 0–50 ng/ml SDF-1 (R&D Systems, Minneapolis, MN, USA). In brief, cells were suspended in 0.5% BSA Phenol Red-Free RPMI and plated in the upper chambers of an HTS-Transwell-96-well permeable support plate (Corning, Acton, MA, USA) and incubated at 37°C in 5% CO_2_ for 2 h. After 2 h incubation, migrated cells were counted using BD TruCount (BD Biosciences) by flow cytometry.

### Murine Studies

#### Mice

Female NOD/ShiLtJ (NOD) or non-obese diabetic mice (NOD) which is the commonly used model for autoimmune T1D studies, NOD.FVB-Tg (CAG-luc,-GFP)L2G85Chco/FathJ (Luciferase NOD) mice which exhibit a widespread expression of the two cell tracers eGFP and firefly luciferase directed by the CAG promoter allowing thus an easily tracking of the cells and NOD.CgTg (TcraBDC2.5,TcrbBDC2)1Doi/DoiJ (BDC2.5 NOD) mice which has the particularity to carry a rearranged TCR a and eight genes from a diabetogenic T cell clone, BDC2.5 and is commonly used *in vitro* autoimmune assays; were purchased from the Jackson Laboratory (Bar Harbor, ME, USA). All mice were housed under specific pathogen-free conditions at an Association for Assessment and Accreditation of Laboratory Animal Care International-accredited facility at BCH. Institutional guidelines and protocols were approved and adhered to the Institutional Animal Care and Use Committee.

#### Murine Regulatory KL Cell Modulation

Murine bone marrow KL (Lineage^−^c-Kit^+^) cells were isolated using magnetic beads and MACS^®^ separation columns (Miltenyi Biotec, San Diego, CA, USA) and ~2 × 10^5^ cells were plated in a U-bottomed 96-well plate with 200 µl of stem cell medium, Stemspan-SFEMII (STEMCELL Technologies, Cambridge, MA, USA) and PGE2 (PromoKine, PromoCell Gmbh, Germany) was added at day 0 and day 1, at a concentration of 10 µM.

#### Flow Cytometric Analysis and Intracellular Cytokine Staining

Flow cytometry was performed to analyze surface expression markers of PGE2-modulated HSPCs and dmPGE2 (16, 16-dimethyl PGE2)-modulated HSPCs. Anti-mouse PD-L1, PD-L2, PD-1, CD40, CD80, CD86, CD4, CD8, Ly-6G (Gr-1), B220, CD3, CXCR4, CCR2, CCR4, CCR5, CCR6, CCR7, CCR8, CXCR3, IL-4, IL-10, and IFN-γ were purchased from BD Biosciences, eBioscience (San Diego, CA, USA) and BioLegend. The following antibodies corresponded to isotype controls for the murine antibodies above: PE mouse IgG1, κ isotype ctrl, Armenian hamster IgG; APC mouse IgG2b, κ isotype ctrl, Armenian hamster IgG. Cells were subjected to FACS analysis and were run on a FACSCalibur™ (Becton Dickinson). Data were analyzed using FlowJo software version 8.7.3 (Treestar).

#### Intracellular Staining for Flow Cytometry

Naïve CD4^+^CD25^−^ T cells (5 × 10^5^) were isolated from BDC2.5 TCR tg mice with a negative selection strategy using a CD4^+^ CD25^+^ Regulatory T cell isolation kit (Miltenyi Biotec) and were stimulated with BDC2.5 peptides and CD11c^+^ DCs (2.5 × 10^5^) previously isolated using CD11c^+^ mAb-coated microbeads. DCs were added at a 1:2 ratio to T cells and were cocultured with PGE2-modulated KL cells (PGE2-KL) at ratios of 1:1, 5:1, and 10:1 (ratio of T cells to PGE2-KL) or alone (controls) or with untransduced KL cells for 24 h in RPMI 10% FBS in a humidified incubator at 37°C, 5% CO_2_. After incubation, cells were collected, washed, and plated in RPMI 10% FBS, then stimulated with 50 ng/ml PMA (Sigma Aldrich, St. Louis, MO, USA), 750 ng/ml ionomycin (Sigma Aldrich) and the protein transport inhibitor BD GolgiStop (6 μl per 6 ml of RPMI as recommended by the manufacturer, BD Biosciences) for 5 h in a humidified incubator at 37°C, 5% CO_2_. After incubation, cells were collected, washed, stained for surface marker CD4 APC, followed by washing and permeabilization using the BD Cytofix/Cytoperm Kit (BD Biosciences) and staining with anti-mouse IFN-γ (eBioscience). Finally, CD4^+^ IFN-γ^+^ cells were assessed by flow cytometric analysis.

#### Pancreas Digestion and Preparation for Flow Cytometry

Pancreata were collected in ice-cold IMDM medium, cut into small pieces, and digested with Collagenase D for 1 h at 37°C, with DNase I added after 30 min. Digested pancreata were passed through a 70-µm cell strainer to obtain single cell suspensions and then analyzed by flow cytometry. For tracking GFP^+^ cells, biotinylated anti-GFP (BD Biosciences) was used at 20 μg/ml followed by staining with APC-conjugated streptavidin (BD Biosciences).

### Statistical Analysis

Statistical analysis was performed using an unpaired Student’s *t* test. A two-sided value of *P* ≤ 0.05 was considered statistically significant. All graphs were generated using GraphPad Prism software version 5.0b (GraphPad Software, Inc., La Jolla, CA, USA). All statistical tests were performed at the 5% significance level.

## Results

### AHSCT Improves β Cell Function in Treated T1D Patients

**Figure 1:**
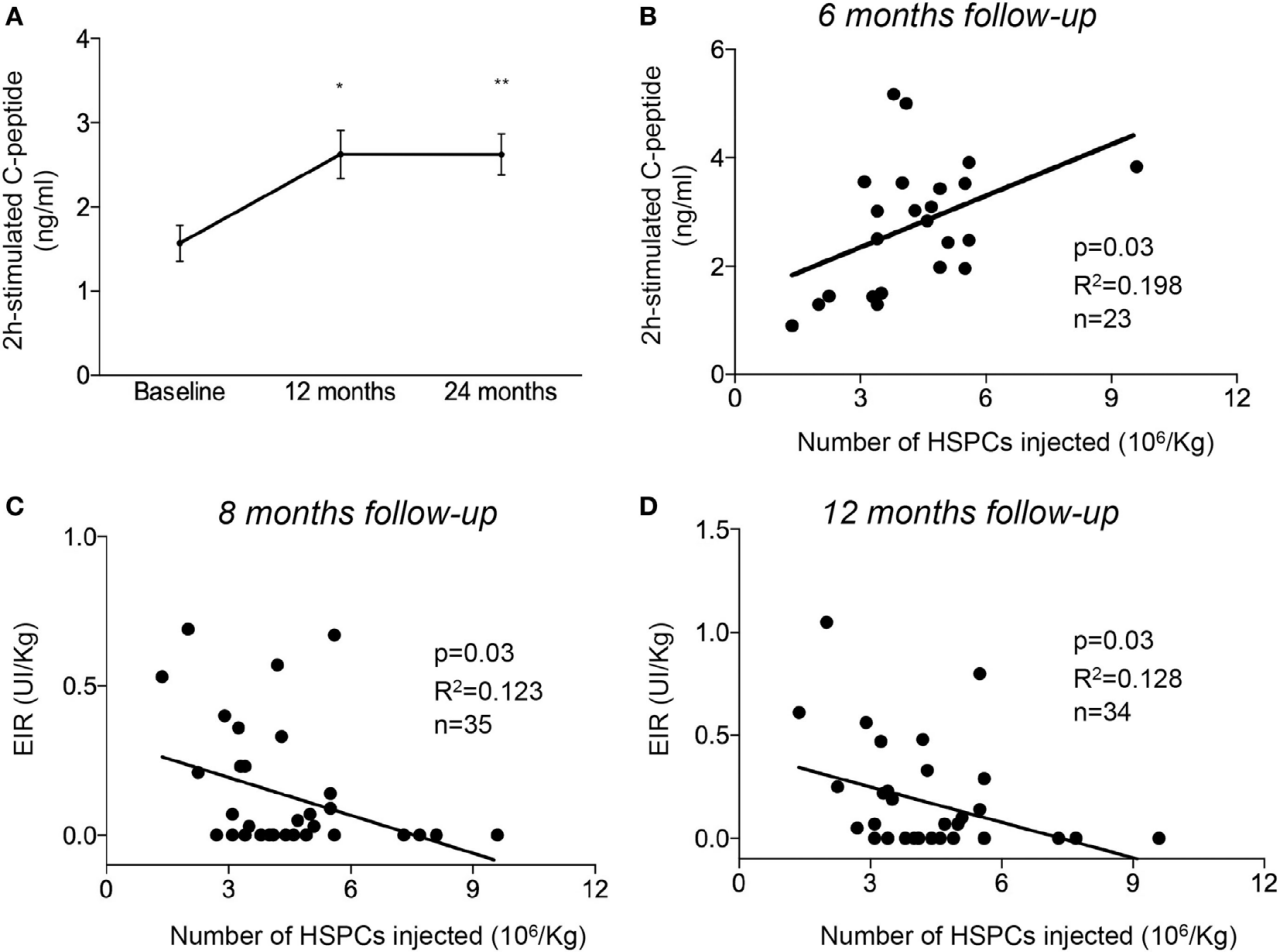
Non-myeloablative AHSCT preserved pancreatic β cell function in patients newly diagnosed with T1D. **(A)** Line graph showing 2 h peak-stimulated C-peptide levels after a mixed meal tolerance test following AHSCT, at 12 and 24 months of follow-up. **(B)** Correlation between the number of HSPCs injected during AHSCT and 2 h peak-stimulated C-peptide levels evaluated at 6 months of follow-up. **(C,D)** Correlation between the number of hematopoietic stem cells (HSPCs) injected during AHSCT and EIR evaluated at 8 months **(C)** and 12 months **(D)** of follow-up. All data are expressed as mean ± SEM. All parameters examined were statistically significantly different when comparing baseline values vs. those at 6, 8, and 12 months. *P < 0.05, **P < 0.001. Abbreviations: AHSCT, autologous hematopoietic stem and progenitor cell transplantation; UI, units of insulin; EIR, exogenous insulin requirement units; HSPCs, hematopoietic stem and progenitor cells; T1D, type 1 diabetes.

Two cohorts consisting of 36 T1D patients were enrolled in the AHSCT program and were also enrolled in three independent clinical trials as previously described (6). All baseline demographic and clinical characteristics of the study population are reported in Table 1. The patient group was predominantly male (27 males and 9 females) with a mean age of 22.4 years and a short history of disease duration (within 6 weeks of diagnosis), confirmed by the presence of autoantibodies to islet peptides [glutamic acid decarboxylase antibodies (anti-GAD) were detected in 86% of patients, while other autoantibodies were detected in 17% of patients]. Most of the patients studied (67%) had no previous history of diabetic ketoacidosis/ketosis. The mean body mass index of patients at diagnosis was 20.7 ± 0.5 (kg/m^2^ ± SEM), and their mean glycated hemoglobin of (HbA1c) was 86.6 ± 6.4 (mmol/mol ± SEM). All patients underwent a stem cell mobilization protocol as previously described (6) with cyclophosphamide (2 g/m^2^) and granulocyte colony-stimulating factor (5–10 µg/kg) daily, beginning the day after cyclophosphamide administration (6). A mean dose of 5.8 ± 0.8 × 10^6^/kg cryopreserved CD34^+^ cells was administered as a single infusion at day 0 (6). All patients showed improvement in β cell function, as revealed by an increase in C-peptide levels over time, which reached a persistent and stable median value >2.5 ng/ml at 12 months of follow-up and lasted until 24 months after treatment (Figure 1A). Interestingly, compared to pre-AHSCT treatment levels, 2 h postprandial peak-stimulated C-peptide levels increased significantly at 6 months post-AHSCT treatment and reached ~2.7 ng/ml at 24 months of follow-up (Figure 1A). Furthermore, a significant positive correlation, albeit weak, was found between the number of CD34^+^ cells infused and C-peptide levels at 6 months after treatment (Figure 1B). Statistical analysis revealed a significant negative correlation between the number of CD34^+^ cells injected during AHSCT and exogenous insulin requirement units evaluated at 8 months (Figure 1C) and 12 months (Figure 1D) of follow-up. T1D patients treated with autologous hematopoietic stem cell transplantation showed an overall improvement in glycometabolic control and maintenance of β cell function.

### PG Library Screening

**Figure 2:**
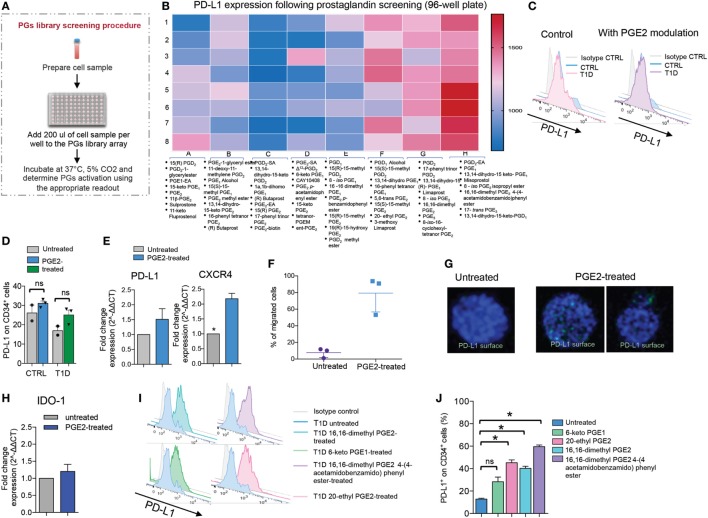
Prostaglandins (PGs) enhance PD-L1 expression on human CD34^+^ cells. **(A,B)** Results of screening of a PG small molecule library tested for the ability to upregulate PD-L1 (MFI) on CD34^+^ cells obtained from T1D patients. The schematic of the experimental design is shown in **(A)**. The 3-color coding shown in **(B)** represents lowest PD-L1 MFI values (blue), median PD-L1 MFI values (pink), and highest PD-L1 MFI values (red). **(C,D)** Representative flow cytometric analysis and quantitative bar graph of PD-L1 expression on CD34^+^ cells from T1D patients pre- and post-pharmacologic modulation with PGE2 as compared to CD34^+^ cells obtained from healthy controls. **(E)** PD-L1 and CXCR4 expression (mRNA) fold change was quantified for CD34^+^ cells pre- and post-modulation with PGE2. **(F)** Migration assay using CD34^+^ cells pre- and post-modulation with PGE2. **(G)** Confocal imaging of CD34^+^ cells pre- and post-modulation with PGE2, showing DAPI (in blue) and PD-L1 (in green) staining. 63× magnification. Scale bar, 50 µm. **(H)** IDO-1 expression (mRNA) fold change was quantified for CD34^+^ cells pre- and post-modulation with PGE2. **(I,J)** Representative flow cytometric analysis **(I)** and quantitative bar graph **(J)** of PD-L1 expression on CD34^+^ cells from T1D patients pre- and post-pharmacologic modulation with four small molecule PGE2 agonists. All data are expressed as mean ± SEM. *P < 0.05, **P < 0.001. Abbreviations: MFI, mean fluorescence intensity; T1D, type 1 diabetes; PGE2, prostaglandin E2.

Prostaglandin E2 has been described as a small molecule known to enhance the homing and engraftment of HSPCs. We therefore sought to screen all known types of PGs using the Prostaglandin Screening Library II, which contains 64 small molecules. We first screened each small molecule contained in the library for its capacity to upregulate PD-L1 in human CD34^+^ cells isolated from T1D patients (Figures 2A,B). CD34^+^ cells isolated from T1D patients were cultured in StemSpan SFEMII and pulsed with each PG small molecule contained in the aforementioned library at a concentration of 10 µM at 24 and 48 h. Using FACS analysis, we calculated the mean fluorescence intensity of PD-L1 expression following treatment with each small molecule used to modulate CD34^+^ cells as compared to untreated/unmodulated CD34^+^ cells (Figure 2B). We generated a heat map depicting the degree to which each PG small molecule affected PD-L1 expression on CD34^+^ cells (Figure 2B), thus allowing us to evaluate PG candidates. Based on its ability to modulate PD-L1 expression, we selected PGE2 as a candidate for further study; in addition, PGE2 has been described in the literature and has been tested in clinical trials as a potential therapy for enhancing HSPC engraftment following cord blood transplantation (21). Isolated CD34^+^ cells from healthy control patients (CTRL) and from T1D patients were cultured in StemSpan SFEMII and pulsed with 10 µM of PGE2 at 24 and 48 h. We first tested the effect of pharmacological modulation with PGE2 by FACS analysis, and our data revealed a slight increase in PD-L1 expression, although not significant, in cultured CD34^+^ cells from T1D and healthy control patients, with the latter showing a higher percentage of PD-L1 expression as compared to cells from T1D patients (Figures 2C,D). This pattern was further confirmed by confocal imaging, in which PD-L1 surface expression was upregulated in PGE2-treated CD34^+^ cells as compared to untreated (Figure 2G). Similar results were obtained by RT-PCR, which demonstrated a slight increase (although not significant) in PD-L1 expression in CD34^+^ cells upon modulation with PGE2 (Figure 2E). Notably, RT-PCR showed a twofold increase of CXCR4 gene expression, a protein required for the homing of HSPCs, in PGE2-treated CD34^+^ cells as compared to untreated (Figure 2E). We therefore sought to perform a migration assay in order to assess the homing properties of PGE2-treated CD34^+^ cells (Figure 2F). Our data confirmed a substantial increase in the homing potential of PGE2-treated CD34^+^ cells (Figure 2F). The expression of another relevant immunoregulatory protein, IDO-1, remained unchanged post-pharmacologic modulation with PGE2 (Figure 2H). We next selected the four PG small molecules [16,16-dimethyl PGE2, 16,16-dimethyl PGE2 4-(4-acetamidobenzamido) phenyl ester, 6-keto PGE1 and 20-ethyl PGE2] that showed the strongest capacity to upregulate PD-L1, based on the results obtained from library screening (Figures 2I,J). By a PGE2-analog and two competitive inhibitors of 15-hydroxy PG dehydrogenase, which possess a prolonged half-life *in vivo* [16,16-dimethyl PGE2, 16,16-dimethyl PGE2 4-(4-acetamidobenzamido) phenyl ester and 20-ethyl PGE2]. FACS analysis of PD-L1 protein expression following pharmacologic modulation with these four PGs (Figures 2I,J) demonstrated robust upregulation of PD-L1 expression. These results were further confirmed by RT-PCR, which showed a marked upregulation of PD-L1 mRNA following pharmacological modulation with 16,16-dimethyl PGE2, 16,16-dimethyl PGE2 4-(4-acetamidobenzamido) phenyl ester and 20-ethyl PGE2 (data not shown).

### PGE2 Highly Augments PD-L1 Expression in Human HSPCs When Supplemented With Hematopoietic Cytokines

**Figure 3:**
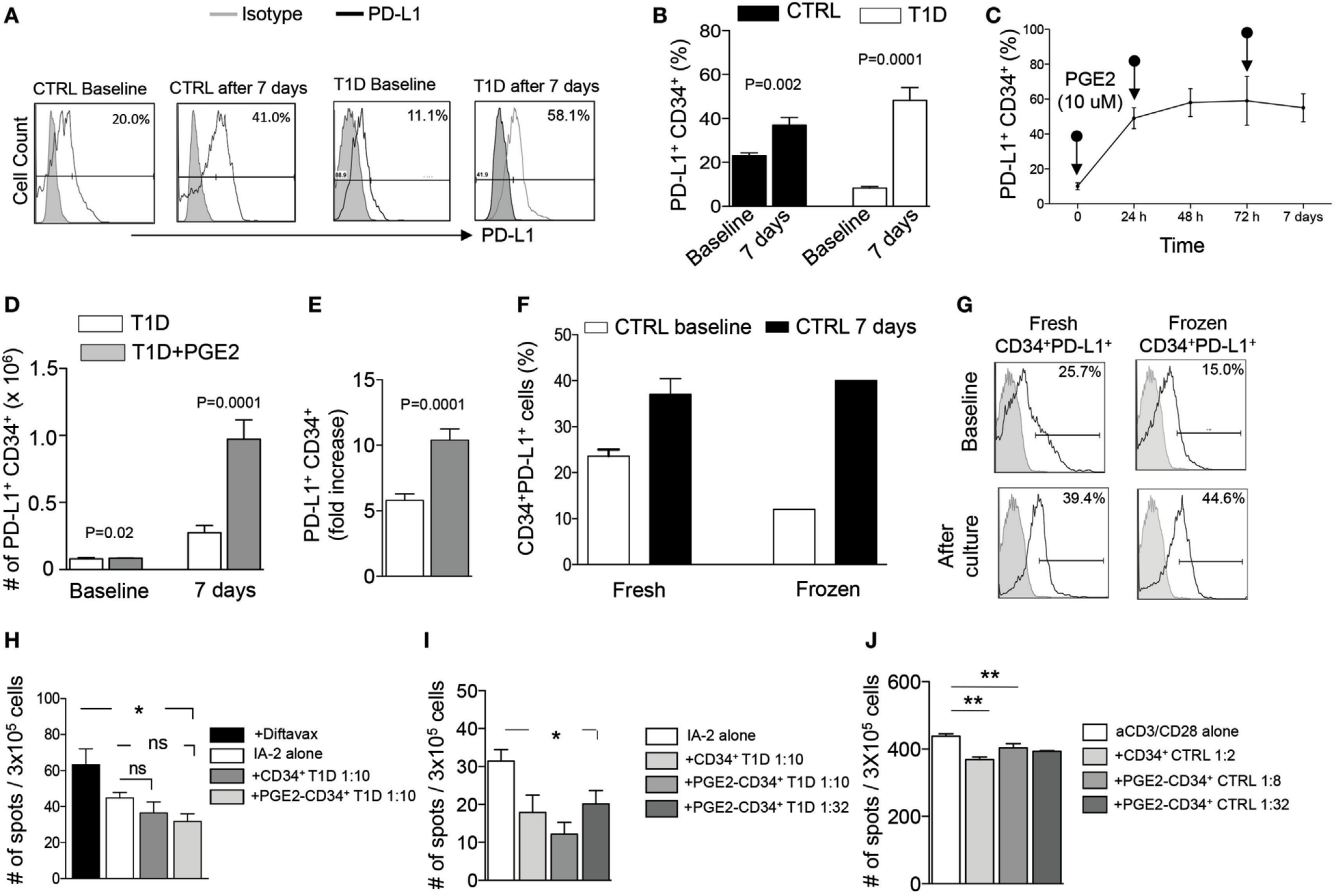
Effects of human PGE2-modulated and cytokine-treated CD34^+^ cells. **(A,B)** Sustained and robust upregulation of PD-L1 upon culture for 7 days with PGE2 and a cocktail of cytokines (heparin, human SCF, human TPO, human FGF-1, IGFBP2, and Angptl3) on CD34^+^ cells obtained from T1D patients as compared to those from healthy controls. **(C)** Effect of PGE2 pulsing on CD34^+^ cells cultured for 0, 24, 72 h and 6 days on PD-L1 expression on CD34^+^ cells. **(D,E)** Bar graphs showing an increase in the number of PD-L1^+^ CD34^+^ cells and fold increase in cell number after 7 days of culture with PGE2 supplemented with cytokines. **(F,G)** Upregulation of PD-L1 expression following culture with PGE2 (shown as percentage) on CD34^+^ cells was not altered by the freeze/thawing process. **(H)** PGE2-modulated CD34^+^ cells abrogate the IFN-γ autoimmune response to insulin-associated 2 (I-A2) autoantigen *in vitro*, as measured *via* the quantification of IFN-γ-producing cells in an Elispot assay; Diftavax refers to a vaccine including immunization against tetanus toxoid, difteria, and hemophilus. **(I)** PGE2-modulated CD34^+^ cells and PGE2-modulated CD34^+^ cells cultured for 7 days in STFIA media abrogate the IFN-γ autoimmune response toward insulin-associated 2 (I-A2) autoantigen *in vitro*, as measured *via* the quantification of IFN-γ-producing cells in an Elispot assay, even when added at low dose. **(J)** PGE2-modulated CD34^+^ cells abrogate the anti-CD3/CD28-stimulated PBMC response *in vitro* as measured *via* the quantification of IFN-γ-producing cells in an Elispot assay. Data are expressed as mean ± SEM. Data are representative of at least two independent experiments. *P < 0.05; **P < 0.01. Abbreviations: SCF, stem cell factor; TPO, thrombopoietin; hFGF-1, human fibroblast growth factor 1; IGFBP2, insulin-like growth factor-binding protein 2; Angptl3, angiopoietin-like 3; PBMCs, peripheral blood mononuclear cells; PGE2, prostaglandin E2; T1D, type 1 diabetes.

In order to improve the strategy used for HSPC expansion and to enhance the function of PGE2-modulated HSPC, we added hematopoietic cytokines (SCF, TPO, FGF-1, IGFBP-2, and Angptl-3 proteins) known as a potent cocktail for HSPC maintenance, into our established culture conditions (22). Isolated CD34^+^ cells (HSPCs) obtained from T1D patients and from healthy controls were cultured using StemSpan SFEMII supplemented with the aforementioned human stem cell growth factors (STFIA medium) and pulsed with PGE2 (10 µM) at 24, 96 h and at 7 days at 37°C 5% CO_2_. PD-L1^+^ HSPCs were then quantified by FACS analysis at different time points post-culture. After 7 days, a ~5-fold increase in the percentage of PD-L1^+^CD34^+^ cells was evident in human HSPCs obtained from T1D, with a similar albeit much less pronounced increase in the percentage of PD-L1^+^CD34^+^ cells obtained from healthy control patients (~2-fold increase) (Figures 3A–E). We next determined whether freezing/cryopreservation has any effect on PD-L1 expression, by comparing freshly isolated HSPCs with frozen HSPCs, after 7 days of culture using STFIA media pulsed with PGE2 (Figures 3F,G). We observed sustained and conserved PD-L1 expression pre- and post-cryopreservation, suggesting that storage of HSPCs has no detrimental impact on their *ex vivo* expansion.

### PGE2-Modulated Human HSPCs Abrogate the Autoimmune Response *Ex Vivo*

To study the *ex vivo* immunoregulatory effects of PGE2 modulation as well as whether cytokine treatment enhances these effects, we performed an autoimmune assay using unmodulated CD34^+^ cells, PGE2-modulated CD34^+^ cells, or PGE2-modulated HSPCs cultured for 7 days in STFIA medium. CD34-depleted PBMCs were cocultured with control CD34^+^ cells (unmodulated), PGE2-modulated CD34^+^ cells, or STFIA medium-cultured PGE2-modulated human CD34^+^ cells in the presence of insulin-associated autoantigen-2 (I-A2) peptide at different cell ratios (1:2, 1:8, and 1:32 CD34^+^ cells to PBMCs), and the number of IFN-γ-producing cells was quantified using an ELISPOT assay (Figures 3H,I). Interestingly, addition of PGE2-modulated human CD34^+^ cells resulted in a significant decrease in the number of IFN-γ-producing cells (Figure 3H), suggesting that PGE2-modulated CD34^+^ cells are endowed with immunoregulatory activity *ex vivo*. Addition of PGE2-modulated CD34^+^ cells cultured for 7 days in STFIA medium showed a further abrogation of the autoimmune response, and this effect was observed even when cells were added at a very low ratio (1:32) to PBMCs (Figure 3I). The effects of PGE2-modulated CD34^+^ cells cultured for 7 days in STFIA media were further confirmed in a nonautoimmune-specific anti-CD3/anti-CD28 assay (Figure 3J).

### Murine PGE2-Modulated HSPCs Abrogate the Autoimmune Response *In Vitro*

**Figure 4:**
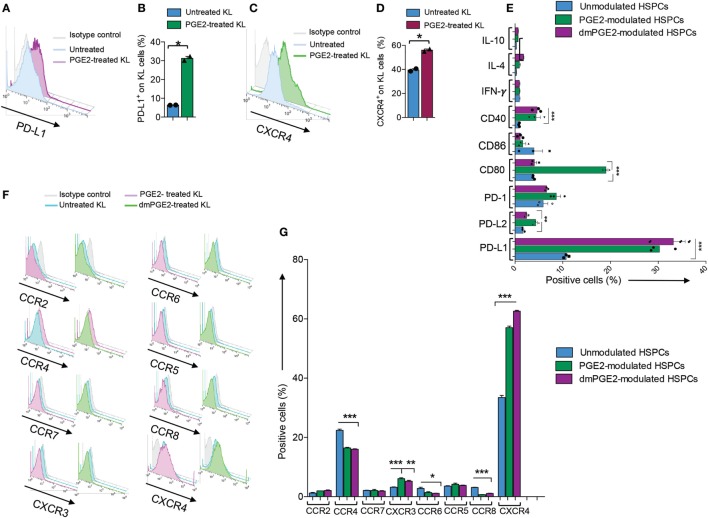
Profile of murine KL cells. **(A,B)** Representative flow cytometric analysis and quantitative bar graph of PD-L1 expression on Lineage^−^c-kit^+^ (KL) cells from NOD mice pre- and post-pharmacologic modulation with PGE2. **(C,D)** Representative flow cytometric analysis and quantitative bar graph of CXCR4 expression on KL cells from NOD mice pre- and post-pharmacologic modulation with PGE2. **(E)** Quantitative bar graph of flow cytometric expression of positive and negative costimulatory molecules (CD40, CD80, CD86, PD-L1, PD-L2, and PD-1) and of select pro-inflammatory and anti-inflammatory cytokines (IFN-γ, IL-10, and IL-4) in PGE2-modulated KL cells (PGE2-KL) from NOD mice as compared to those modulated with a selected PGE2 clinical grade agonist (dmPGE2) and compared to unmodulated KL cells isolated from the bone marrow of normoglycemic NOD mice. **(F)** Flow cytometric expression of selected chemokine receptors (CXCR4, CCR2, CCR4, CCR5, CCR6, CCR7, CCR8, and CXCR3) in PGE2-KL from NOD mice as compared to those modulated with a selected PGE2 clinical grade agonist (dmPGE2) and to unmodulated KL cells isolated from the bone marrow of normoglycemic NOD mice. **(G)** Quantitative bar graph of flow cytometric expression of chemokines receptors (CCR2, CCR4, CCR5, CCR6, CCR7, CCR8, CXCR3, and CXCR4) in PGE2-KL from NOD mice as compared to those modulated with a selected PGE2 clinical grade agonist (dmPGE2) and compared to unmodulated KL cells isolated from the bone marrow of normoglycemic NOD mice. Data are expressed as mean ± SEM. Data are representative of at least two independent experiments. *P < 0.05; **P < 0.01; ***P < 0.001. Abbreviations: KL, Lineage^−^c-kit^+^ cells; PGE2, prostaglandin E2.

**Figure 5:**
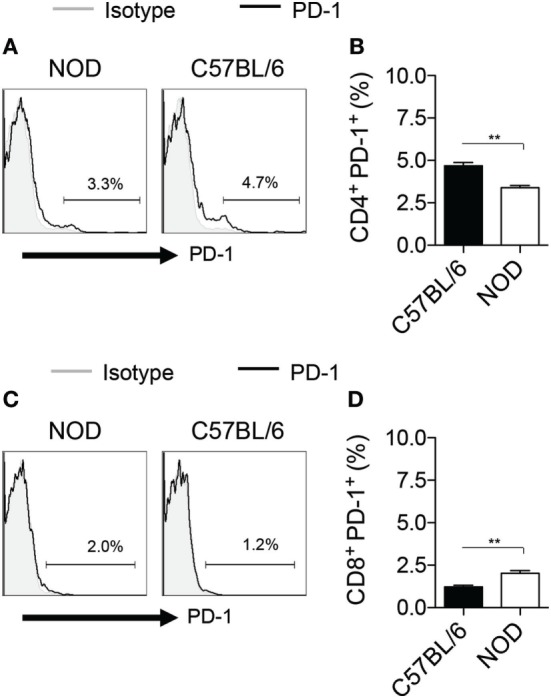
PD-1 expression on murine T cells. **(A,B)** Representative flow cytometric analysis **(A)** and quantitative bar graph **(B)** for PD-1^+^CD4^+^ T cells isolated from splenocytes of NOD mice as compared those from C57BL/6 mice. **(C,D)** Representative flow cytometric analysis **(C)** and quantitative bar graph **(D)** for PD-1^+^CD8^+^ T cells isolated from splenocytes of NOD mice as compared those from C57BL/6 mice.

**Figure 6:**
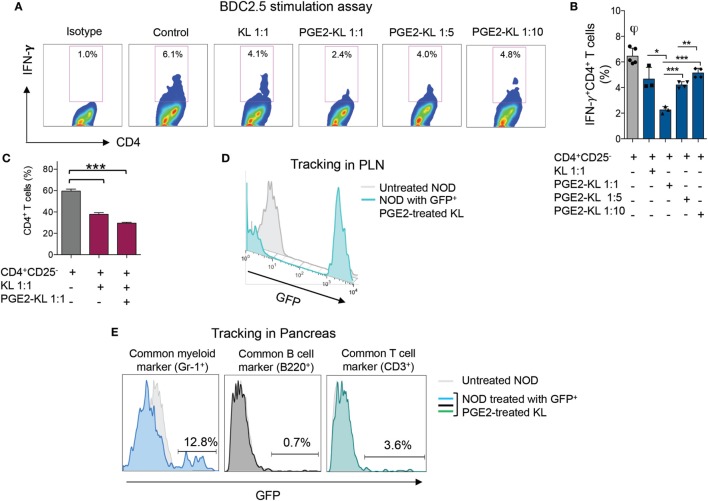
Effects of murine KL cells. **(A,B)** Representative flow cytometric analysis **(A)** and quantitative bar graph **(B)** for IFN-γ^+^CD4^+^ T cells isolated from NOD-BDC2.5 TCR Tg mice and stimulated with BDC2.5 peptide in the presence of DCs (Control) or upon coculture with unmodulated KL or with PGE2-KL (at different ratios). **(C)** Quantitative bar graph of the percentage of CD4^+^ T cells upon coculture with KL or PGE2-KL. **(D)** Representative flow cytometric analysis of GFP^+^PGE2-KL in the PLN of treated NOD mice following 24 h of treatment with GFP^+^PGE2-KL, demonstrating that they traffic to the PLN following adoptive transfer into NOD mice. **(E)** Representative flow cytometric analysis of GFP^+^PGE2-KL in the pancreas of NOD mice 24 h post-infusion with GFP^+^PGE2-KL, demonstrating the surface phenotype of GFP^+^ cells. Abbreviations: KL, Lineage^−^c-kit^+^ cells; PGE2-KL, PGE2-modulated KL cells; HSPCs, hematopoietic stem and progenitor cells; GFP, green fluorescent protein; PGE2, prostaglandin E2; DCs, dendritic cells.

We next explored the feasibility of pharmacological modulation of PD-L1 with PGE2 in murine HSPCs. FACS analysis showed an upregulation of PD-L1 post-PGE2 modulation in KL (Lineage^−^c-Kit^+^) cells isolated from bone marrow of NOD mice (Figures 4A,B). Furthermore, we assessed PD-1 on T cells from NOD mice as compared to those from C57BL/6 and our data showed a significant defect in PD-1 expression on CD4 T cells isolated from NOD mice (Figure 5). Interestingly, another protein, CXCR4, primary involved in the homing of HSPCs, was markedly upregulated (Figures 4C,D). We then analyzed the expression of costimulatory molecules, as well as pro-inflammatory and anti-inflammatory cytokines by flow cytometry, which demonstrated upregulation of PD-L2, PD-1, CD80, and CD40 in PGE2-KL as compared to unmodulated KL cells. The expression of these molecules in KL cells modulated with dmPGE2 (known as 16, 16-dimethyl PGE2, a molecule which exerts a prolonged effect *in vivo* as compared to PGE2) was similarly upregulated (Figure 4E). Moreover, we explored the chemokine receptor profile of PGE2-KL as compared to dmPGE2-modulated KL cells and unmodulated KL cells in order to assess which chemokines are potentially involved in the homing of PGE2-modulated and dmPGE2-modulated KL cells. Consistent with the results in Figure 4C, CXCR4 was the most expressed chemokine in both groups of KL cells treated with PGE2 and dmPGE2 (Figures 4F,G). We next explored the immunoregulatory properties of PGE2-KL in an autoimmune setting *in vitro*. PGE2-KL generated from normoglycemic NOD mice were cocultured at ratios of 1:1, 1:5, and 1:10 (KL cells to T cells) with CD11c^+^ DCs and BDC2.5 transgenic CD4^+^CD25^−^ T cells in the presence of BDC2.5 peptide. Quantification by flow cytometry revealed a pronounced and significant decrease in the percentage of IFN-γ^+^CD4^+^ T cells when PGE2-KL were added to the assay as compared to when unmodulated KL cells were used (Figures 6A,B). Indeed, PGE2-KL exerted a robust immunoregulatory effect even if added at low ratios (1:5 PGE2-treated KL cell to T cells). A less pronounced effect was observed when unmodulated KL were added to the assays. Interestingly, the percentage of activated CD4^+^CD25^+^ T cells declined upon coculture with KL or PGE2-KL (Figure 6C). PGE2-modulated HSPCs are thus endowed with immunoregulatory properties and are capable of abrogating the autoimmune response *in vitro*.

### Adoptively Transferred Murine PGE2-Modulated HSPCs Traffic to Inflamed Areas

To examine the trafficking properties of GFP^+^ PD-L1-expressing KL cells in an *in vivo* inflammatory setting, we performed a set of tracking experiments in NOD mice. Following infusion of GFP^+^ KL cells extracted from the bone marrow of Luciferase NOD-GFP mice and treated with PGE2 as previously described, the pancreas and pancreatic draining lymph nodes (PLN) of NOD mice were harvested at 24 h. GFP^+^ cells were quantified in the aforementioned tissues by flow cytometry and were detectable in the PLN (Figure 6D) and in the pancreata of NOD mice (Figure 6E). The phenotype of the GFP^+^ cells after adoptive transfer and after homing to the pancreata of NOD mice showed Gr-1 expression, indicative of myeloid lineage, while very few cells were CD3^+^ (Figure 6E). These GFP^+^ PD-L1-expressing cells might probably interact with autoreactive CD4 and CD8 T cells.

## Discussion

The prospect of successful cell therapy has recently gained greater footing in the medical landscape in the past 2 years with the arrival of many cell-based products. Recently, many AHSCT-related clinical trials have demonstrated a beneficial effect in the treatment of several autoimmune diseases, and AHSCT is now considered one of the few therapies capable of reversing T1D in humans (6, 14, 25–27). In our study, we observed preservation of β cell function following AHSCT, as most patients included in our study population exhibited a sustained and adequate postprandial C-peptide response. The majority of these patients achieved and maintained peak-stimulated C-peptide levels higher than 0.6 ng/ml for at least 2 years of follow-up. Sustained C-peptide secretion is known to be associated with reduced prevalence (~30%) of hypoglycemic events and with a slower progression of diabetes complications, as reported by the DCCT Trial (28). Several patients also experienced reversal of the disease or a decrease in the exogenous insulin daily requirement. Although these are very encouraging results, many investigators have reported various complications and adverse effects associated with AHSCT in T1D patients, primarily related to the effects of immunosuppression (6). Some patients experience only temporary remission, and thus achieving prolonged remission of the disease remains the foremost goal for future clinical trials. Recently, much progress has been made with regard to the identification of small molecules and growth factors capable of both enhancing HSPC proliferation (15, 16) and further expanding the immunomodulatory subsets of HSPCs, in order to capitalize on their immunosuppressive properties. Interestingly, a screening study performed in zebrafish embryos showed that PGE2 enhances HSPC expansion and facilitates HSPC engraftment after bone marrow transplantation (21). Investigating and determining the effects of *ex vivo* modulation of HSPCs with PGE2 in an autoimmune setting may provide insight with regard to how to robustly enhance their immunoregulatory properties. Our screening results performed on ~64 known PGs allowed us to select four PGs, which are analogs to PGE2 and which we show induce relatively high upregulation of PD-L1 expression on human CD34^+^ cells. We therefore sought to test the ability of PGE2-modulated HSPCs to affect the autoimmune response *in vitro*. Compared to unmodulated HSPCs, HSPCs overexpressing PD-L1 successfully abrogated the human autoimmune response *in vitro*. Next, we sought to explore whether refining the *ex vivo* culture approach by including a cocktail of potent cytokines important for HSPC maintenance and extending the length of culture to 7 days could enhance the effects observed. Importantly, this approach remarkably enhanced the immunoregulatory properties of HSPCs and induced more pronounced PD-L1 expression. This expression appeared to be stable, unaffected by the freeze/thaw process, and resulted in a potent abrogation of the autoimmune response by modulated HSPCs, even when added at a very low ratio to T cells. Paralleling the human data, these preclinical murine studies also confirmed that PGE2-modulated HSPCs similarly exhibited immunoregulatory effects, as they markedly abrogated CD4-restricted autoimmune responses *in vitro. In vivo* tracking studies suggested that PGE2-modulated HSPCs home to the inflamed pancreas and PLN of NOD mice, most likely due to their substantial expression of CXCR4 (9). Based on the data herein, *ex vivo* expansion strategies with PGE2 combined with hematopoietic cytokines could generate a novel immunoregulatory HSPC-based approach potentially useful in the treatment of autoimmune T1D, without the detrimental effect of immunosuppressive agent toxicity, which is observed with standard immunotherapy. The recent discovery that a pre-established suicide genetic system may control survival and prevent toxicity of HSPCs undergoing *ex vivo* expansion will implement their use in clinical settings, allowing for easier manipulation of HSPCs and for a cell therapy-based approach in immune-mediated disorders (29).

## Ethics Statement

This study was carried out in accordance with the recommendations of Institutional Review Board committee approval of San Raffaele Hospital, Milan-Italy (P2X7-T1D/01 and PD-L1-T1D/01) and BCH (3851).

## Author Contributions

MN designed and performed experiments, analyzed data, and wrote the paper; FD, AMM, SF, EC-L, FR, TL, AE, EA, AM, EG, AD, VU, CL, CM, EG, and MM performed experiments; GC, GB, MS, and GZ coordinated research; PF designed the study, wrote and edited the paper.

## Conflict of Interest Statement

The authors declare that the research was conducted in the absence of any commercial or financial relationships that could be construed as a potential conflict of interest. The reviewer, DC and the handling Editor declared their shared affiliation.
