# Supplementary material for: Prostaglandin E2 Stimulates the Expansion of Regulatory Hematopoietic Stem and Progenitor Cells in Type 1 Diabetes
Source: Front Immunol. 2018 Jun 19;9:1387. doi: 10.3389/fimmu.2018.01387 (PMC6018202; doi:10.3389/fimmu.2018.01387)
Supplement: Supplementary file 1 [file table_1.DOCX]

**(A This Supplemental Information file includes:**

**SUPPLEMENTAL DATA:**

**SUPPLEMENTAL TABLE S1**

**Supplemental Tables**

**Table S1.** The composition of the Prostaglandins Screening Library II (Cayman Chemicals, Ann Arbor, MI).

| Item Catalog # | Item name | Row | Column |
| --- | --- | --- | --- |
| 10118 | 15(R)-Prostaglandin D2 | A | 1.00 |
| 10140 | Prostaglandin E2-1-glyceryl ester | B | 1.00 |
| 10192 | Prostaglandin D2 serinol amide | C | 1.00 |
| 10193 | Prostaglandin E2 serinol amide | D | 1.00 |
| 12000 | Prostaglandin D1 | E | 1.00 |
| 12002 | Prostaglandin D1 Alcohol | F | 1.00 |
| 12010 | Prostaglandin D2 | G | 1.00 |
| 12012 | Prostaglandin D2 Ethanolamide | H | 1.00 |
| 12015 | Prostaglandin D2-1-glyceryl ester | A | 2.00 |
| 12410 | 11-deoxy-11-methylene Prostaglandin D2 | B | 2.00 |
| 12610 | 13,14-dihydro-15-keto Prostaglandin D2 | C | 2.00 |
| 12650 | Δ12-Prostaglandin D2 | D | 2.00 |
| 12720 | 15(R)-15-methyl Prostaglandin D2 | E | 2.00 |
| 12730 | 15(S)-15-methyl Prostaglandin D2 | F | 2.00 |
| 12810 | 17-phenyl trinor Prostaglandin D2 | G | 2.00 |
| 13010 | Prostaglandin E1 | H | 2.00 |
| 13012 | Prostaglandin E1 Ethanolamide | A | 3.00 |
| 13020 | Prostaglandin E1 Alcohol | B | 3.00 |
| 13050 | 1a,1b-dihomo Prostaglandin E1 | C | 3.00 |
| 13260 | 6-keto Prostaglandin E1 | D | 3.00 |
| 13360 | 8-iso Prostaglandin E1 | E | 3.00 |
| 13610 | 13,14-dihydro Prostaglandin E1 | F | 3.00 |
| 13630 | 13,14-dihydro-15(R)-Prostaglandin E1 | G | 3.00 |
| 13650 | 13,14-dihydro-15-keto Prostaglandin E1 | H | 3.00 |
| 13680 | 15-keto Prostaglandin E1 | A | 4.00 |
| 13730 | 15(S)-15-methyl Prostaglandin E1 | B | 4.00 |
| 13745 | (R)-Butaprost | C | 4.00 |
| 13747 | CAY10408 | D | 4.00 |
| 13750 | 16,16-dimethyl Prostaglandin E1 | E | 4.00 |
| 13770 | 16-phenyl tetranor Prostaglandin E1 | F | 4.00 |
| 13810 | Limaprost | G | 4.00 |
| 13820 | Misoprostol | H | 4.00 |
| 14010 | Prostaglandin E2 | A | 5.00 |
| 14011 | Prostaglandin E2 methyl ester | B | 5.00 |
| 14012 | Prostaglandin E2 Ethanolamide | C | 5.00 |
| 14053 | Prostaglandin E2 p-acetamidophenyl ester | D | 5.00 |
| 14054 | Prostaglandin E2 p-benzamidophenyl ester | E | 5.00 |
| 14210 | 5-trans Prostaglandin E2 | F | 5.00 |
| 14350 | 8-iso Prostaglandin E2 | G | 5.00 |
| 14352 | 8-iso Prostaglandin E2 isopropyl ester | H | 5.00 |
| 14510 | 11β-Prostaglandin E2 | A | 6.00 |
| 14650 | 13,14-dihydro-15-keto Prostaglandin E2 | B | 6.00 |
| 14710 | 15(R)-Prostaglandin E2 | C | 6.00 |
| 14720 | 15-keto Prostaglandin E2 | D | 6.00 |
| 14725 | 15(R)-15-methyl Prostaglandin E2 | E | 6.00 |
| 14730 | 15(S)-15-methyl Prostaglandin E2 | F | 6.00 |
| 14750 | 16,16-dimethyl Prostaglandin E2 | G | 6.00 |
| 14753 | 16,16-dimethyl Prostaglandin E2 p-(p-acetamidobenzamido) phenyl ester | H | 6.00 |
| 14765 | Sulprostone | A | 7.00 |
| 14770 | 16-phenyl tetranor Prostaglandin E2 | B | 7.00 |
| 14810 | 17-phenyl trinor Prostaglandin E2 | C | 7.00 |
| 14840 | tetranor-PGEM | D | 7.00 |
| 14910 | 19(R)-hydroxy Prostaglandin E2 | E | 7.00 |
| 14940 | 20-ethyl Prostaglandin E2 | F | 7.00 |
| 14990 | Prostaglandin E3 | G | 7.00 |
| 14995 | 17-trans Prostaglandin E3 | H | 7.00 |
| 16783 | 11-keto Fluprostenol | A | 8.00 |
| 10006045 | (R)-Butaprost (free acid) | B | 8.00 |
| 10006987 | Prostaglandin E2-biotin | C | 8.00 |
| 10008294 | ent-Prostaglandin E2 | D | 8.00 |
| 10008385 | Prostaglandin D2 methyl ester | E | 8.00 |
| 10008445 | 3-methoxy Limaprost | F | 8.00 |
| 10009278 | 8-iso-16-cyclohexyl-tetranor Prostaglandin E2 | G | 8.00 |
| 10010425 | 13,14-dihydro-15-keto Prostaglandin D1 | H | 8.00 |
